# Supplementary material for: Variant-specific inflation factors for assessing population stratification at the phenotypic variance level
Source: Nat Commun. 2021 Jun 9;12:3506. doi: 10.1038/s41467-021-23655-2 (PMC8190158; doi:10.1038/s41467-021-23655-2)
Supplement: Supplementary file 1 — Supplementary Information [file 41467_2021_23655_MOESM1_ESM.pdf]

Variant-Specific Inflation Factors for Assessing Population Stratification at the Phenotypic Variance Level: Supplementary Information

Tamar Sofer et al.

Supplementary Note 1: Estimated distribution of inflation factors 1
Supplementary Note 2: Comparison of variant inflation/deflation categories between BMI and HGB analyses.. 2
Supplementary Note 3: Acknowledgements of participating studies 2

Supplementary Note 1: Estimated distribution of inflation factors

To demonstrate the potential values observed when estimating inflation factors using N=10,000 independent test statistics (e.g., from simulation replicates), we performed the following simulation study:

1. Repeat 1,000 times:
    a. Sample 10,000 χ_1^2 random variable with centrality parameter 0, because this is the distribution of a test statistic of the association of variant-outcome association under the null of association. Denote this vector of simulated test statistics by $\boldsymbol{t} = (t_1, \dots, t_N)^T$
    b. Compute the "estimated inflation factor" as the median of the test statistics $median(t_1, \dots, t_N)$, divided by the 0.5 quantile of the χ_1^2 distribution, denote by λ_{sim}.
2. Visualize the distribution of the 1,000 λ_{sim} using a histogram and using a figure mimicking Figure 1 from the main manuscript.

Figure S1: Distribution of inflation factors based on 10,000 independent test statistics under the null.

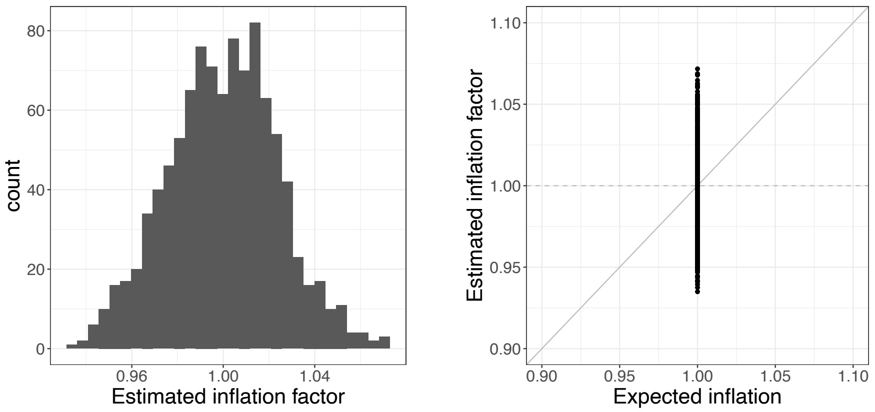

Supplementary Note 2: Comparison of variant inflation/deflation categories between BMI and HGB analyses

For all variants analyzed in both the BMI and HGB analyses (i.e., having MAC of at least 20 in both analyses), we computed categorized each of them, in each of the analyses, as "Inflated", "Deflated", or "Approximately no inflation", based on the computed λ_{vs} (as described in the main manuscript). Figure S2 below demonstrate the distributions and overlap in inflation/deflation categories in the two analyses, separately for common variants (defined as having MAF>0.05), and for rare variants (defined as having MAF<0.05). Because MAF may differ when computed across different set of individuals, for categorizing variants to common and rare we computed MAF over the pooled set of individuals who participated in the BMI analysis.

Figure S2: Distribution and overlap in inflation/deflation categories of variants used in the HGB and BMI analyses.

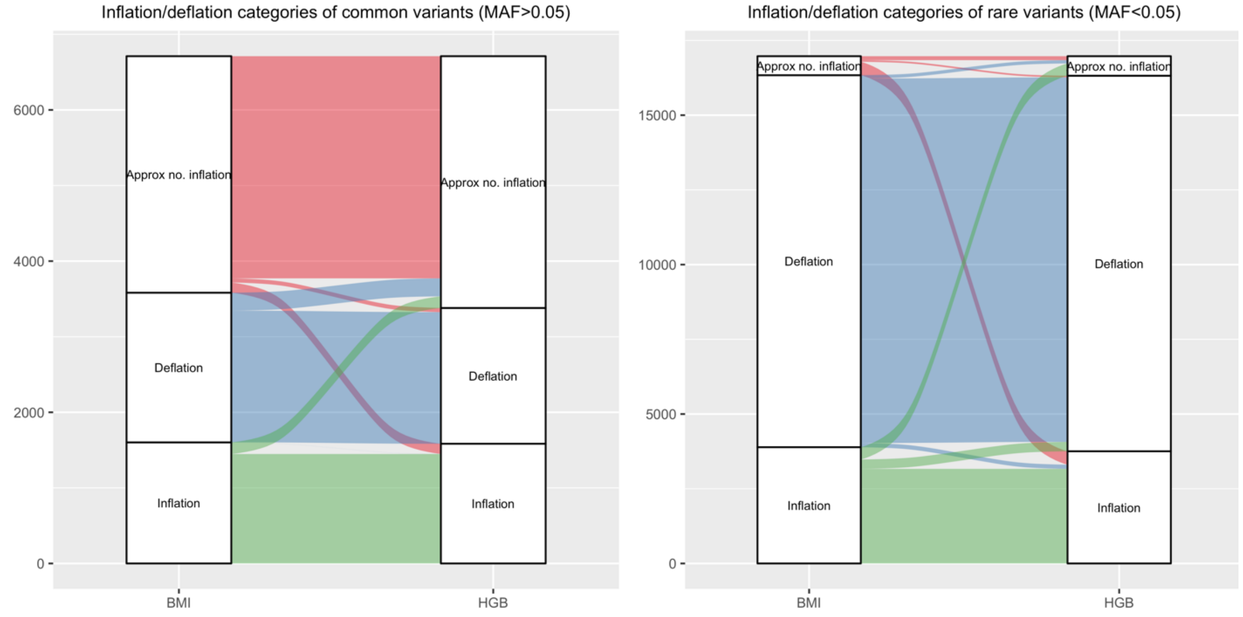

The Y-axis provides the number of variants counted, in thousands.

Supplementary Note 3: Acknowledgements of participating studies

Framingham Heart Study

The Framingham Heart Study (FHS) acknowledges the support of contracts NO1-HC-25195 and HHSN268201500001I from the National Heart, Lung and Blood Institute and grant supplement R01 HL092577-06S1 for this research. We also acknowledge the dedication of the FHS study participants without whom this research would not be possible. FHS participated in both the hemoglobin and BMI analysis.

Jackson Heart Study
JHS is a longitudinal community-based study designed to assess the causes of the high prevalence of cardiovascular disease among AAs in the Jackson, Mississippi metropolitan area [11]. During the baseline examination period (2000-2004) 5,306 self-identified AAs were recruited from urban and rural areas of the three counties (Hinds, Madison and Rankin) that comprise the Jackson, Mississippi metropolitan area. Participants were between 35 and 84 years old with the exception of a nested family cohort, where those ≥21 years old were eligible All participants included in analyses provided written informed consent for genetic studies. Approval was obtained from the institutional review board of the University of Mississippi Medical Center (UMMC). Data on participants' health behaviors, medical history, and medication use were collected at baseline and subjects underwent venipuncture, allowing for assessment of complete blood cell counts and other measures at UMMC (Beckman-Coulter, [12]). JHS participated in both the hemoglobin and BMI analysis.

The Jackson Heart Study (JHS) is supported and conducted in collaboration with Jackson State University (HHSN268201300049C and HHSN268201300050C), Tougaloo College (HHSN268201300048C), and the University of Mississippi Medical Center (HHSN268201300046C and HHSN268201300047C) contracts from the National Heart, Lung, and Blood Institute (NHLBI) and the National Institute for Minority Health and Health Disparities (NIMHD). The authors also wish to thank the staffs and participants of the JHS.

The Amish study
We gratefully acknowledge our Amish liaisons, research volunteers, field workers and Amish Research Clinic staff and the extraordinary cooperation and support of the Amish community without which these studies would not have been possible. The Amish studies are supported by grants and contracts from the NIH, including U01 HL072515, U01 HL84756, U01 HL137181 and P30 DK72488. The Amish study participated in both the hemoglobin and BMI analysis.

The Genetic Epidemiology of Asthma in Costa Rica – Asthma Costa Rica Cohort
Costa Rica is a Hispanic population with asthma prevalence 24%. This study originated in 1997 with the recruitment of both extended pedigrees and trios in the central valley of Costa Rica. The study participated in the BMI analysis.

Cleveland Family Study
The Cleveland Family Study (CFS, [13]) was designed to examine the genetic basis of sleep apnea in 2,534 African-American and European-American individuals from 356 families. CFS participated in the BMI analysis.

COPDGene
COPDGene is a cross sectional prospective cohort enrolled between January 2008 and June 2011 at 21 Clinical Centers. The study goals were to characterize smokers with and without COPD, using spirometry, six minute walk, medical history, respiratory symptoms (modified ATS respiratory questionnaire), respiratory medications, quality of life and inspiratory and

expiratory chest CT scans, and to perform epidemiological and genetic studies of these COPD-related phenotypes. Participants from COPDGene who consented for data use for method development participated in the BMI analysis.

The COPDGene project was supported by Award Number U01 HL089897 and Award Number U01 HL089856 from the National Heart, Lung, and Blood Institute. The content is solely the responsibility of the authors and does not necessarily represent the official views of the National Heart, Lung, and Blood Institute or the National Institutes of Health. The COPDGene project is also supported by the COPD Foundation through contributions made to an Industry Advisory Board comprised of AstraZeneca, Boehringer Ingelheim, GlaxoSmithKline, Novartis, Pfizer, Siemens and Sunovion.

TOPMed acknowledgements

Whole genome sequencing (WGS) for the Trans-Omics in Precision Medicine (TOPMed) program was supported by the National Heart, Lung and Blood Institute (NHLBI). WGS for “NHLBI TOPMed: Genetics of Cardiometabolic Health in the Amish” (phs000956) was performed at the Broad Institute of MIT and Harvard (3R01HL121007-01S1). WGS for “NHLBI TOPMed: Cleveland Family Study - WGS Collaboration” (phs000954) was performed at the University of Washington Northwest Genomics Center (3R01HL098433-05S1, HHSN268201600032I). WGS for “NHLBI TOPMed: Genetic Epidemiology of COPD Study” (phs000951) was performed at the University of Washington Northwest Genomics Center (3R01HL089856-08S1), and at the Broad Institute of MIT and Harvard (HHSN268201500014C). WGS for “NHLBI TOPMed: The Genetic Epidemiology of Asthma in Costa Rica - Asthma in Costa Rica cohort” (phs000988) was performed at the University of Washington Northwest Genomics Center (3R37HL066289-13S1, HHSN268201600032I). WGS for “NHLBI TOPMed: Framingham Heart Study” (phs000974) was performed at the Broad Institute of MIT and Harvard (3R01HL092577-06S1, 3U54HG003067-12S2). WGS for “NHLBI TOPMed: Jackson Heart Study” (phs000964) was performed at the University of Washington Northwest Genomics Center (HHSN268201100037C).

Centralized read mapping and genotype calling, along with variant quality metrics and filtering were provided by the TOPMed Informatics Research Center (3R01HL-117626-02S1; contract HHSN268201800002I). Phenotype harmonization, data management, sample-identity QC, and general study coordination were provided by the TOPMed Data Coordinating Center (3R01HL-120393-02S1; contract HHSN268201800001I). We gratefully acknowledge the studies and participants who provided biological samples and data for TOPMed.
