# Supplementary material for: Variant-specific inflation factors for assessing population stratification at the phenotypic variance level
Source: Nat Commun. 2021 Jun 9;12:3506. doi: 10.1038/s41467-021-23655-2 (PMC8190158; doi:10.1038/s41467-021-23655-2)
Supplement: Supplementary file 3 — Description of Additional Supplementary Files [file 41467_2021_23655_MOESM3_ESM.pdf]

Description of Additional Supplementary Files

File Name: Supplementary Software 1
Description: A tutorial for computing variant-specific inflation factors
