# Supplementary material for: Variant-specific inflation factors for assessing population stratification at the phenotypic variance level
Source: Nat Commun. 2021 Jun 9;12:3506. doi: 10.1038/s41467-021-23655-2 (PMC8190158; doi:10.1038/s41467-021-23655-2)
Supplement: Supplementary file 5 — Reporting Summary [file 41467_2021_23655_MOESM5_ESM.pdf]

nature research

Corresponding author(s): Tamar Sofer

Last updated by author(s): 2020/08/21

Reporting Summary

Nature Research wishes to improve the reproducibility of the work that we publish. This form provides structure for consistency and transparency in reporting. For further information on Nature Research policies, see our Editorial Policies and the Editorial Policy Checklist.

Statistics

For all statistical analyses, confirm that the following items are present in the figure legend, table legend, main text, or Methods section.

n/a	Confirmed	
☐	☒	The exact sample size (*n*) for each experimental group/condition, given as a discrete number and unit of measurement
☐	☒	A statement on whether measurements were taken from distinct samples or whether the same sample was measured repeatedly
☐	☒	The statistical test(s) used AND whether they are one- or two-sided<br>*Only common tests should be described solely by name; describe more complex techniques in the Methods section.*
☐	☒	A description of all covariates tested
☐	☒	A description of any assumptions or corrections, such as tests of normality and adjustment for multiple comparisons
☒	☐	A full description of the statistical parameters including central tendency (e.g. means) or other basic estimates (e.g. regression coefficient) AND variation (e.g. standard deviation) or associated estimates of uncertainty (e.g. confidence intervals)
☒	☐	For null hypothesis testing, the test statistic (e.g. *F*, *t*, *r*) with confidence intervals, effect sizes, degrees of freedom and *P* value noted<br>*Give P values as exact values whenever suitable.*
☒	☐	For Bayesian analysis, information on the choice of priors and Markov chain Monte Carlo settings
☒	☐	For hierarchical and complex designs, identification of the appropriate level for tests and full reporting of outcomes
☒	☐	Estimates of effect sizes (e.g. Cohen's *d*, Pearson's *r*), indicating how they were calculated

Our web collection on statistics for biologists contains articles on many of the points above.

Software and code

Policy information about availability of computer code

Data collection: no software was used

Data analysis: We used the open access software R version 4.0.0, the R/Bioconductor package GENESIS version 2.18.0, the MetaCor R package available on GitHub https://github.com/tamartsi/MetaCor, and for plotting, we used the package ggplot2 version 3.3.0. We deposited code that we developed in a GitHub repository https://github.com/tamartsi/Variant_specific_inflation.

Sequencing data were prepared using the following software: Illumina RTA2 (Real Time Analysis 2), bcl2fastq v2.15.0, bcl2fastq2 v2.16.0.10, HiSeq Control Software v3.1.26, BWA-MEM v0.7.8, and Bamutil v1.0.9.

For manuscripts utilizing custom algorithms or software that are central to the research but not yet described in published literature, software must be made available to editors and reviewers. We strongly encourage code deposition in a community repository (e.g. GitHub). See the Nature Research guidelines for submitting code & software for further information.

Data

Policy information about availability of data

All manuscripts must include a data availability statement. This statement should provide the following information, where applicable:

- Accession codes, unique identifiers, or web links for publicly available datasets
- A list of figures that have associated raw data
- A description of any restrictions on data availability

TOPMed whole genome sequencing data are available, from TOPMed Freeze 5b and Freeze 8, on dbGaP by application to each of the studies used in this manuscript. Phenotypes can also be obtained through application to dbGaP. Study dbGaP accessions are: phs000956 (Amish); phs000954 (CFS); phs000951 (COPDGene); phs000988 (CRA); phs000974 (FHS); phs000964 (JHS).

Field-specific reporting

Please select the one below that is the best fit for your research. If you are not sure, read the appropriate sections before making your selection.

- [x] Life sciences
- [ ] Behavioural & social sciences
- [ ] Ecological, evolutionary & environmental sciences

For a reference copy of the document with all sections, see nature.com/documents/nr-reporting-summary-flat.pdf

Life sciences study design

All studies must disclose on these points even when the disclosure is negative.

Sample size	This manuscript reports a secondary data analysis. We used all available TOPMed Freeze 4 data with the phenotypes of interest and appropriate consent.
Data exclusions	No exclusions: all individuals with phenotype values and appropriate consent were included.
Replication	This is a methodological paper, that do not report specific results. Therefore, we do not report any replication.
Randomization	There was no randomization because the data do not represent a trial, but rather an observational study.
Blinding	No blinding, because there was no trial/experiment, we analyzed observational data.

Reporting for specific materials, systems and methods

We require information from authors about some types of materials, experimental systems and methods used in many studies. Here, indicate whether each material, system or method listed is relevant to your study. If you are not sure if a list item applies to your research, read the appropriate section before selecting a response.

Materials & experimental systems

n/a	Involved in the study	
x		Antibodies
x		Eukaryotic cell lines
x		Palaeontology and archaeology
x		Animals and other organisms
	x	Human research participants
x		Clinical data
x		Dual use research of concern

Methods

n/a	Involved in the study	
x		ChIP-seq
x		Flow cytometry
x		MRI-based neuroimaging

Human research participants

Policy information about studies involving human research participants

Population characteristics	9,807 individuals participated in the analysis of BMI, and 7,596 participated in the analysis of hemoglobin concentration. Across the participating cohorts, mean ages were 18 to 53. Mean BMI varied from 20.8 to 32.7, and percentages of males varied from 43.4% to 58.9%.
Recruitment	Multiple studies participated in the analyses in this manuscript. Individuals in these studies are not always representatives of the general population (for example, participants in the CRA Costa Rica study were enriched for Asthma, and participants in the CFS were enriched for Obstructive Sleep Apnea), but we do not expect this to affect the results in the present analyses, because we are not reporting any specific associations, but rather overall patterns. Information on all TOPMed studies, including study design, is available here: https://www.nhlbiwgs.org/group/project-studies .<br><br>Recruitment to the Amish study:<br>The Amish Complex Disease Research Program includes a set of large community-based studies focused largely on cardiometabolic health carried out in the Old Order Amish (OOA) community of Lancaster, Pennsylvania (http://medschool.umaryland.edu/endocrinology/amish/research-program.asp). The OOA population of Lancaster County, PA immigrated to the Colonies from Western Europe in the early 1700's. There are now over 30,000 OOA individuals in the Lancaster area, nearly all of whom can trace their ancestry back 12-14 generations to approximately 700 founders. Investigators at the University of Maryland School of Medicine have been studying the genetic determinants of cardiometabolic health in this population since 1993. To date, over 7,000 Amish adults have participated in one or more of our studies.<br><br>CFS study:<br>The CFS is a genetic epidemiological study of 352 rigorously phenotyped families ascertained through probands with OSA identified through Cleveland, OH area sleep centers, neighborhood controls, and the spouses and first and second degree relatives of probands.<br><br>COPDGene:<br>COPDGene (also known as the Genetic Epidemiology of COPD Study) is an NIH-funded, multicenter study. A study population of more than 10,000 smokers (1/3 African American and 2/3 non-Hispanic White) has been characterized with a study protocol including pulmonary function tests, chest CT scans, six minute walk testing, and multiple questionnaires.<br><br>Recruitment to the CRA Costa Rica (CAMP) study:<br>From February 2001 to August 2008, questionnaires were sent to the parents of 16,912 children (ages 6-14 years) enrolled in 140 Costa Rican schools; 9,180 (54.3%) questionnaires were returned. Children were eligible for the study if they had asthma (physician-diagnosed asthma and ≥ 2 respiratory symptoms or asthma attacks in the prior year) and a high probability of having ≥ 6 great-grandparents born in the Central Valley of Costa Rica (as determined by the study genealogist on the basis of the paternal and maternal last names of each of the child's parents).<br><br>FHS:<br>The original FHS cohort were 5,209 men and women between the ages of 30 and 62 from Framingham, MA. In 1971, the study enrolled a second-generation cohort (Framingham Offspring Study) -- 5,124 of the original participants' adult children and their spouses.<br><br>JHS:<br>The Jackson Heart Study (JHS) https://www.jacksonheartstudy.org/jhsinfo/ is a large, community-based, observational study whose participants were recruited from urban and rural areas of the three counties (Hinds, Madison and Rankin) that make up the Jackson, MS metropolitan statistical area (MSA). Participants were enrolled from each of 4 recruitment pools: random, 17%; volunteer, 30%; currently enrolled in the Atherosclerosis Risk in Communities (ARIC) Study, 31% and secondary family members, 22%. Recruitment was limited to non-institutionalized adult African Americans 35-84 years old, except in a nested family cohort where those 21 to 34 years of age were also eligible.
Ethics oversight	As public datasets available by application from the NHI dbGaP repository, all participants provided informed consent to their parent studies at their recruitment site.<br>Amish: All study protocols were approved by the institutional review board at the University of Maryland Baltimore. Informed consent was obtained from each study participant.<br>CFS: Cleveland Family Study was approved by the Institutional Review Board (IRB) of Case Western Reserve University and Mass General Brigham (formerly Partners HealthCare). Written informed consent was obtained from all participants.<br>COPDGene: All COPDGene participants provided written informed consent, and the study was approved by the Institutional Review Boards of the participating clinical centers.<br>CAMP: All CAMP study parents provided informed consent and participants provided consent/assent and the study was approved by the Institutional Review Boards of all 8 clinical sites (Denver, St. Louis, San Diego, Boston, Baltimore, Albaquerque, Toronto, Seattle) and the Data Coordinating Center (Baltimore).<br>FHS: The Framingham Heart Study was approved by the Institutional Review Board of the Boston University Medical Center. All study participants provided written informed consent.<br>JHS: The JHS study was approved by Jackson State University, Tougaloo College, and the University of Mississippi Medical Center IRBs, and all participants provided written informed consent.

To participate in any given TOPMed analysis, PIs of each participating study approved the manuscript concept, the use of data, after verifying that the data usage agrees with the specific consent, and the manuscript was further approved by TOPMed publication committee. Thus, both the data was collected with appropriate consent, and the data was used in accordance with this consent.

The secondary data analysis provided here was approved by the Partners Human Research IRB and by the Institutional Review Board of the University Washington.

Note that full information on the approval of the study protocol must also be provided in the manuscript.
